# A case of anterior spinal cord syndrome in a patient with unruptured thoracic aortic aneurysm with a mural thrombus

**DOI:** 10.1186/s12872-018-0786-4

**Published:** 2018-03-05

**Authors:** Nilukshana Yogendranathan, H. M. M. T. B. Herath, W. D. Jayamali, Anne Thushara Matthias, Aruna Pallewatte, Aruna Kulatunga

**Affiliations:** National hospital, Colombo, Sri Lanka

**Keywords:** Anterior spinal cord infarction, Thoracic aortic aneurysm

## Abstract

**Background:**

Spinal cord infarction is an uncommon condition. Anterior cord syndrome present with paraparesis or quadriparesis with sparing of vibration and proprioceptive senses. The common causes of anterior cord syndrome are aortic dissection and aortic surgical interventions. Spontaneous unruptured nondissected aortic aneurysms with intramural thrombus can rarely cause anterior cord infarctions.

**Case presentation:**

We report a case of anterior spinal cord syndrome due to aneurysm of the thoracic aorta with a mural thrombus. A 64 year old male presented with sudden onset paraparesis with a sensory level at T1 with preserved sense of proprioception and vibration. The MRI panspine revealed increased T2 intensity in the anterior portion of the spinal cord from C5 to T10 level with characteristic ‘owl eye’ appearance on axial imaging. The CT aortogram detected aneurysmal dilatation of the ascending aortic, arch and descending thoracic aorta with significant intimal irregularities, calcified atherosclerotic plaques and a small mural thrombus.

**Conclusion:**

The possible mechanisms postulated are occlusion of ostia of radicular arteries by the atherosclerotic plaques and mural thrombus or thromboembolism to the anterior spinal artery. Nondissected atherosclerotic aortic aneurysms should be considered in patients presenting with spinal cord infarctions especially in the presence of vascular risk factors and smoking.

## Background

Spinal cord infarction is an uncommon entity caused by numerous pathological conditions. It can present with either acute onset paraparesis or quadriparesis. The presentation depends on the vascular territory involved. The most common type of spinal cord infarction is anterior spinal artery syndrome. The aetiopathogenic mechanisms are aortic dissection/ surgical procedures involving the aorta, intrinsic arterial occlusion of the blood vessels perfusing the spinal cord namely anterior spinal artery or radicular branches, hypotension and venous infarctions.

There had been numerous cases reported of anterior spinal cord syndrome due to aortic dissection and as post procedural complications. However there had been only few case reports of spontaneous spinal cord infarction due to asymptomatic aortic aneurysm with a mural thrombus in the literature.

We present a previously healthy male smoker presenting with anterior spinal cord syndrome, found to be habouring an asymptomatic aneurysm of the arch, ascending and proximal descending aorta with marked atherosclerotic plaques along with a small mural thrombus.

## Case presentation

A 64 year old male presented with sudden onset quadriparesis. He had acute onset pain in both lower limbs which was followed by lower limb weakness. Meantime he had also noticed weakness of both upper limbs. There was no history of preceding back or neck pain. He was also unable to pass urine and faeces. There had not been any history of trauma. He has been ambulant and apparently healthy prior to this illness. He gave a history of smoking of sixteen pack years. There was no past or family history of cardiovascular diseases. There was no history of high risk sexual behaviour.

The patient was averagely built with no features of Marfan syndrome. On examination both the lower limbs had reduced tone and the power was of Medical Research Council {MRC} grade 0/5. Both knee and ankle jerks were absent with equivocal plantar response. The pain and temperature sensory examination revealed a sensory level at T1. However the vibration and proprioception were both spared. The examination of upper limbs revealed weak elbow extension MRC power grade being 4/5. Intrinsic hand muscles power was also reduced to grade 3/5 bilaterally. However biceps and brachioradialis power was 5/5. The biceps and brachioradialis reflexes were normal. The triceps reflex was diminished on right side and absent on the left. Thus a lesion at spinal cord level lower cervical/ upper thoracic C7/T1 was clinically suspected especially of the anterior cord region sparing the dorsal columns. There were no areas of spinal tenderness.

The abdominal examination revealed a palpable distended bladder with relaxed anal sphincter tone. Cardiac and respiratory system examination was essentially normal. The mental status and cognition were unaffected. The patient was immediately catheterized and basic investigations carried out.

**Fig. 1:**
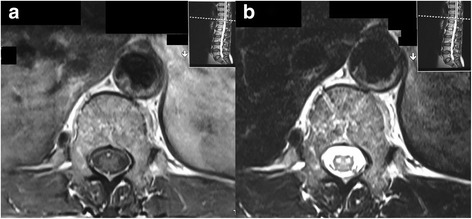
**a** T1 weighted axial image. **b** T2 weighted axial image showing the owl eye appearance depicting the anterior cord ischaemia. The wall thrombus is seen in the aortic aneurysm

**Fig. 2:**
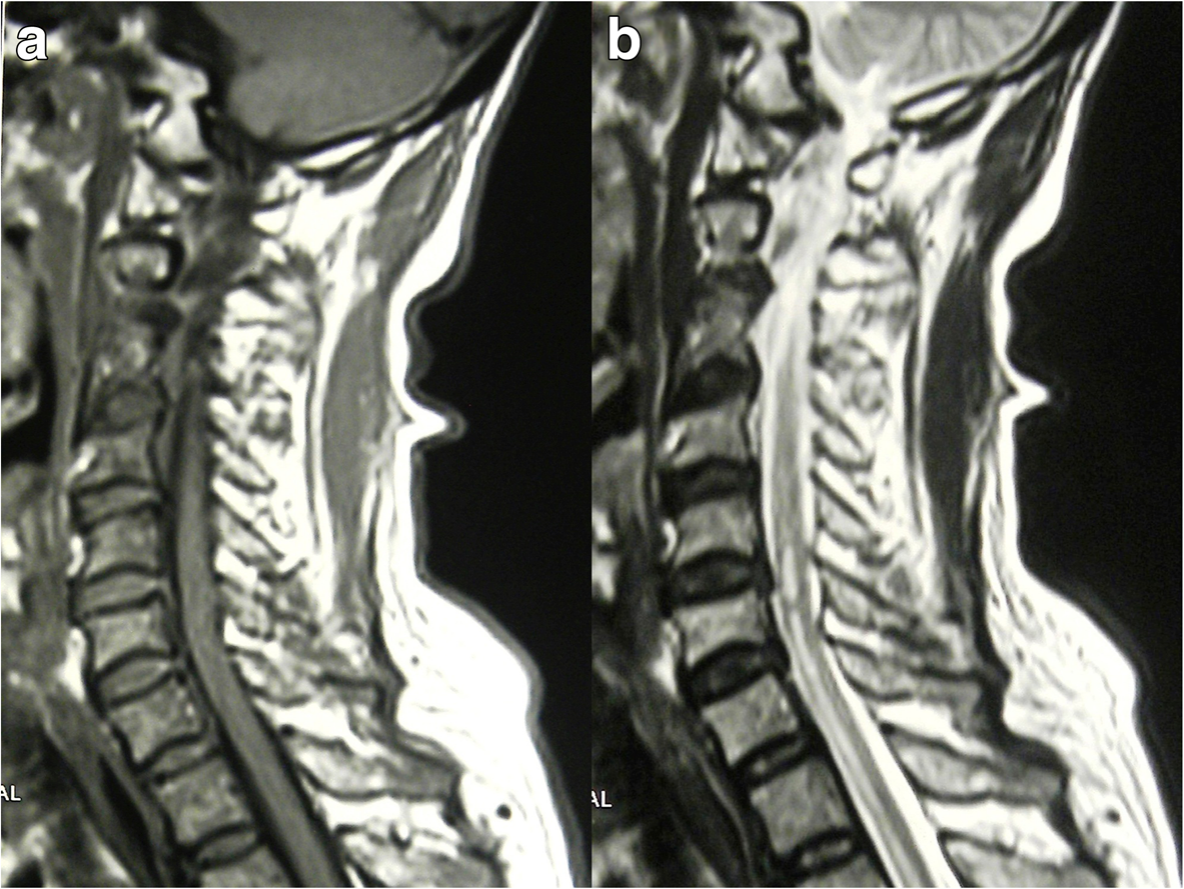
**a** T1 Weighted sagittal image, **b** T2 Weighted sagittal image shows linear hyperintensity along the lower cervical and thoracic cord without cord expansion. This is compatible with ischemia

Magnetic Resonant Imaging (MRI) panspine showed increased T2 signal intensity in the anterior aspect of cervico-dorsal spinal cord extending from C5 to T10 level. High signal areas revealed the characteristic ‘owl eye’ appearance on axial imaging (Figs. 1 and 2). The involved areas showed diffusion restriction on Diffusion Weighted Imaging (DWI). Cervical, thoracic and lumbosacral vertebral bodies and discs were of normal alignment with mild degenerative changes without significant thecal indentation or paravertebral abnormalities. Compressive myelopathy was also excluded. The lower thoracic aorta showed aneurysmal dilatation with a mural thrombus.

**Fig. 3:**
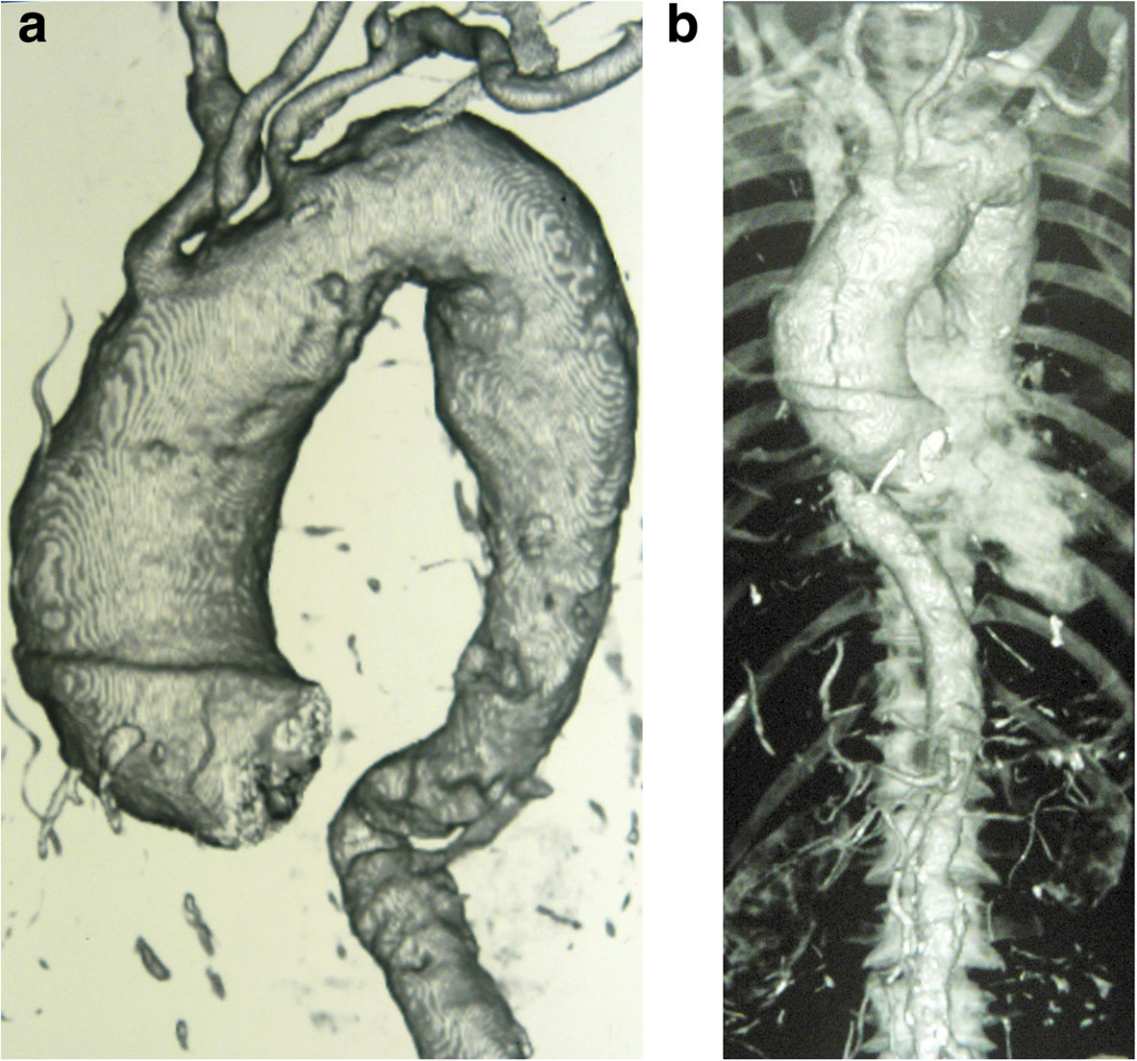
**a** Unfolded aortic arch, b Fusiform dilatation of thoracic aorta

The chest Xray revealed a widened mediastinum. A CT aortogram done thereafter confirmed an aneurysmal dilalatation of the root, ascending, arch and proximal descending thoracic aorta. The abdominal aorta was normal. The diameters of the ascending aorta and arch were 5.6 cm, 5.8 cm respectively (Fig. 3). It can be categorized as Extent 1 according to the Crawford classification of thoracoabdominal aortic aneurysms. There were marked intimal irregularities and calcified atherosclerotic plaques with a small mural thrombus.

The complete blood count, C reactive protein, renal and liver functions were within normal ranges. The ESR was 32 mm/ first hour and Venereal Disease Research Laboratory (VDRL) test was non reactive. HIV 1 and 2 antibodies were negative. Blood and urine cultures were all sterile. The LDL cholesterol was 86 mg/dL and HbA1c was 5.4%. Antinuclear antibody and Antineutrophilic cytoplasmic antibodies were negative. Two Dimentional Echocardiography revealed a mild left ventricular hypertrophy with an ejection fraction of 60% and dilated aortic root of 4 cm in size.

The patient was assessed by both vascular surgical and cardiothoracic teams. However the patient was declining surgical interventions. He was then commenced on rehabilitation and transferred to a dedicated rehabilitation centre.

## Discussion

The patient presented with quadriparesis with a sensory level at T1. The sparing of proprioception and vibration depicts anterior cord involvement. The acute history suggests a possible vascular aetiology. Furthermore the pathognomonic feature of increased T2 signal intensity along with the absence of vertebral pathology, compressive lesions on the MRI point towards anterior spinal cord infarction. Syphilis and autoimmune aetiologies were excluded. CT angiogram revealed marked atherosclerotic plaques in the thoracic aorta with a mural thrombus. The classical MRI features of spinal cord infarction are central T2 hyperintensity without significant cord expansion or contrast enhancement. Diffusion-weighted imaging demonstrated corresponding abnormal restricted diffusion [1].

Three major vessels arising from the vertebral artery supply the spinal cord. The anterior spinal artery which originates from the vertebral artery at the foramen magnum runs along the anterior median sulcus of the spinal cord upto the conus medullaris. It supplies via the sulcal arteries and the peripheral arterial plexus to the central and peripheral parts of the anterior two third of the spinal cord respectively. The posterior spinal arteries arise from the vertebral arteries and travel along the posterior lateral sulci bilaterally and supply the posterior one third of the spinal cord. However the anterior spinal artery has the narrowest diameter in the thoracic segment. The supply of the anterior spinal artery is augmented typically by 6th -10th of the 31 pairs of radicular arteries which arise from the vertebral, intercostals and rarely as direct branches from the aorta. The most prominent thoracic radicular artery known as the artery of Adamkiewicz contributes to the anterior spinal artery between T9 to T12. In comparison the posterior spinal arteries although vary in diameter throughout the course are supported by 10th -23rd of the radicular arteries. The anterior and posterior spinal arteries anastomose in the conus medullaris.

Anterior spinal cord infarction had been a well recognized complication of aortic aneurysmal surgery both open thoracoabdominal aortic aneurysmal repair as well as with thoracic endovascular aortic aneurysm repair (TEVAR) [2, 3]. There are various mechanisms postulated such as injury of the major anterior radicular artery by a clamp applied to the aorta, prolonged hypotension, occlusion of the suprarenal aorta during resection of an abdominal aortic aneurysm [4]. There had also been cases reported of patients with aortic dissection presenting with paraparesis [5]. Cummings and Joo had reported a 63 year old female who has had an aortic dissection compromising the blood supply to the artery of Adamkiewicz presenting with a transient paralysis of lower limbs [6]. Handa et al.; had reported a 72 year old male smoker presenting with acute paraplegia with sensory level at T12/L1 who had been found to have an infrarenal abdominal unruptured aortic aneurysm with extensive mural thrombus. The intraoperative findings of liquid thrombus led to the conclusion of possible massive cholesterol embolism to the distal vasculature [7]. There had been a similar case of a 62 year old Caucasian smoker who had been previously healthy presenting with cauda equina syndrome due to spinal cord infarction and subsequently found to have an abdominal aortic aneurysm with a large mural thrombus which was repaired resulting in substantial improvement of the proximal muscle power [8].

**Fig. 4:**
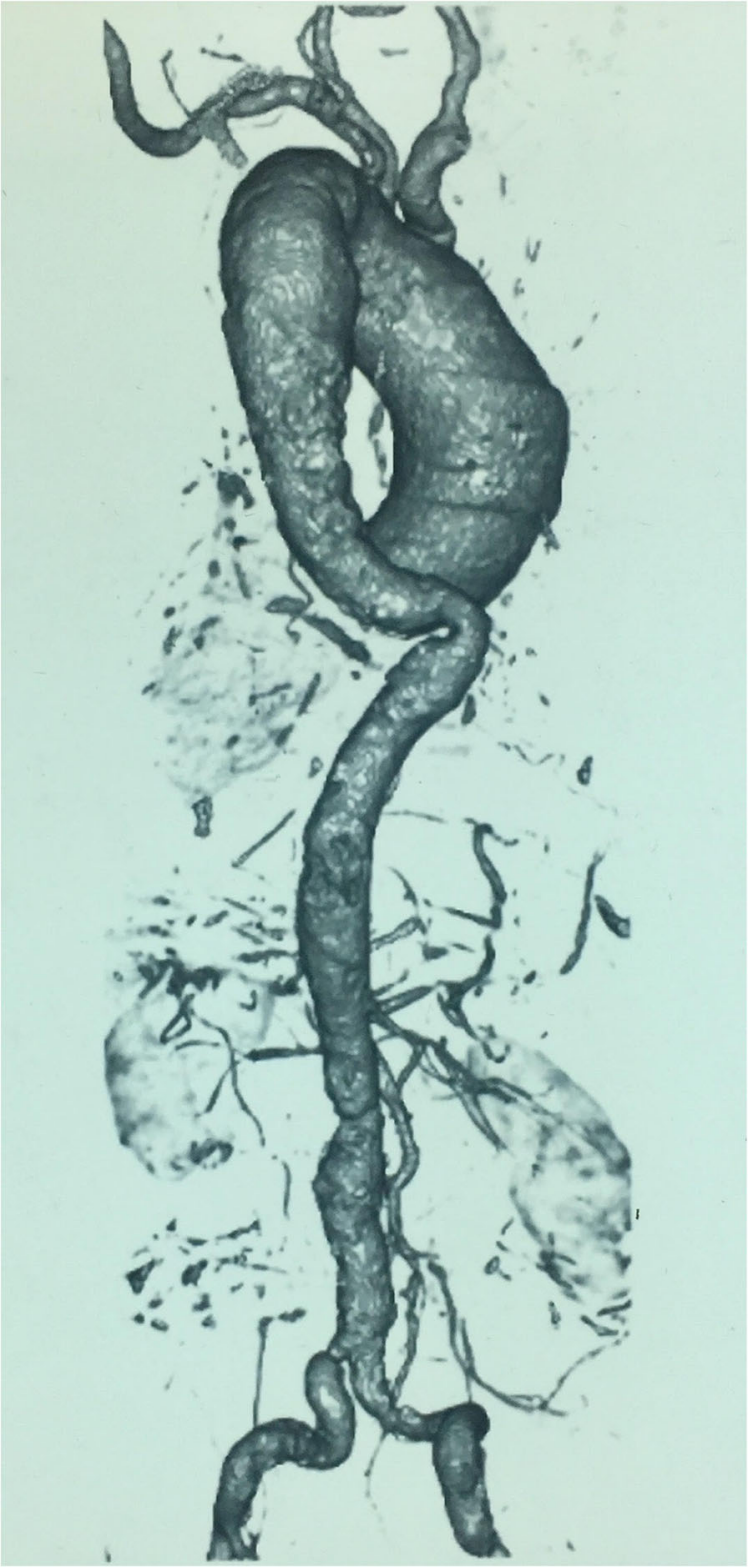
Absence of the origin of segmental branches of thoracic aorta in comparison with the descending aorta is depicted. This could be due to the luminal occlusion of these branches by the mural thrombus

There had only been a single case of anterior cord syndrome reported due to unruptured nondissected thoracoabdominal aortic aneurysm [9]. The abdominal CT revealed a mural thrombus occupying 50% of the lumen of the aorta. The possible mechanisms postulated were detachment of a portion of thrombus of the aortic aneurysm culminating in embolism of the artery of Adamkiewicz or the mural thrombus blocking the origin of the segmental artery that branches out into the artery of Adamkiewicz. In our patient, the possible mechanisms are mural thrombus and the atherosclerotic plaque lesions occluding the ostia of the radicular arteries which may also include the artery of Adamkiewicz at the sites of origin from the aorta (Fig. 4). However a remote possibility of thromboembolism from the mural thrombus in the aneurysm via the vertebral arteries to the anterior spinal artery also should be considered.

The prognosis of spinal cord infarction depends on the aetiology, severity and the regions involved. Our patient did not have any autonomic dysfunction although there was evidence of upper thoracic cord infarction. No treatment modalities including thrombolysis, steroids are of proven benefit to reverse or limit the ischaemic injury caused. The treatment should be aimed to address the aetiology such as surgical repair of vascular malformation or a dissected/ ruptured aortic aneurysm in order to prevent further secondary complications. Nevertheless patients with atherosclerotic risk factors warrant antiplatelet therapy. Our patient was commenced on Aspirin with a statin and was transferred to a rehabilitation centre.

## Conclusion

Spinal cord infarction unlike cerebral infarction is an uncommon entity. Anterior spinal cord syndrome is a well known manifestation of aortic dissection necessitating surgical interventions involving the aorta. Spontaneous unruptured abdominal aortic aneurysms causing anterior cord syndromes due to mural thrombi had only rarely been reported. We report this patient as there had been only one case report of unruptured nondissected thoracic aortic aneurysm with mural thrombus presenting as anterior spinal cord syndrome in the literature upto now.

Furthermore the possibility of unruptured atherosclerotic aortic aneurysm with or without mural thrombi should be considered as a cause in patients presenting with spinal cord infarctions especially males, smokers after exclusion of the common aetiologies such as compressive lesions and vasculitides.

## Abbreviations

MRI: Magnetic Resonance Imaging
